# Emergency Department Presentations and Hospitalisations for Elder Abuse in People Accessing Aged Care Services in Australia: A Retrospective Cross‐Sectional Study

**DOI:** 10.5694/mja2.70172

**Published:** 2026-03-24

**Authors:** Stephanie L. Harrison, Sahar Barmomanesh, Bryan Morden, Maria C. Inacio, Gillian E. Caughey, the ROSA Consumer and Community Advisory Committee

**Affiliations:** ^1^ Registry of Senior Australians Research Centre, Caring Futures Institute, College of Nursing and Health Sciences Flinders University Adelaide South Australia Australia; ^2^ Registry of Senior Australians Research Centre, South Australian Health and Medical Research Institute (SAHMRI) Adelaide South Australia Australia

**Keywords:** aged, domestic violence, elder abuse, health services for the aged

## Abstract

Elder abuse can lead to serious physical injuries and long‐term psychological consequences, but its recognition and documentation in healthcare settings remain limited. This study used linked data from four Australian states to examine elder abuse coded during emergency department presentations and hospitalisations among 965,986 older people assessed for aged care services between 2010 and 2019. Only 580 people (0.06%) had elder abuse coded during an emergency department presentation or hospitalisation, highlighting substantial under‐recognition and under‐reporting in hospital settings.

## 1 Introduction

Elder abuse has serious and lasting psychological, physical, social and economic consequences [1]. The Royal Commission into Aged Care Quality and Safety (2018–2021) described elder abuse in aged care as a ‘source of national shame’ [2]. In 2021, the Serious Incident Response Scheme (SIRS) was introduced to identify and prevent abuse and neglect in aged care. In 2023–2024, 56,955 SIRS incidents were reported in residential aged care and 5005 in home care [3]. In 2022–2023, 355 individuals older than 65 years were hospitalised for injuries related to domestic violence [4]. The extent to which elder abuse leads to emergency department (ED) presentations or hospitalisations among individuals in aged care remains unclear.

## 2 Methods

Using the Registry of Senior Australians (ROSA) National Historical Cohort, this study examined elder abuse coded during ED presentations and hospitalisations among people accessing aged care [5]. We analysed data from 965,986 non‐Indigenous people aged 65–105 years with an aged care eligibility assessment completed between 1 January 2010 and 31 December 2019 in South Australia, New South Wales, Queensland or Victoria. The cumulative incidence of ED presentations and hospitalisations coded for elder abuse using the International Statistical Classification of Diseases and Related Health Problems, 10th revision, Australian Modification [6, 7] (ICD‐10‐AM; Table S1) were estimated for the overall cohort, by year, by state and by aged care type (residential aged care, home care package or neither). Additional analyses examined hospitalisations with injuries related to domestic violence (Methods S1 and Tables S2 and S3). Analyses were conducted using R version 4.3.3. This study is reported according to the Strengthening the Reporting of Observational Studies in Epidemiology (STROBE) guidelines for cross‐sectional studies (Table S4). This study has ethics approvals from the: University of South Australia Human Research Ethics Committee (Ref: 200489); Australian Institute of Health and Welfare Ethics Committee (Ref: EO2022/4/1376); South Australian Department for Health and Wellbeing Human Research Ethics Committee (Ref: HREC/18/SAH/90); and New South Wales Population & Health Services Research Ethics Committee (Ref: 2019/ETH12028).

## 3 Results

The median age of the cohort was 83 years (interquartile range [IQR], 77–88 years); 59.1% (571,139) were female; 43.4% (419,201) lived alone; 67.3% (650,511) lived in a major city and 21.8% (210,713) were living with dementia (Table S5). Median study follow‐up time was 810 days (IQR, 294–1586 days). In the study period, 580 people (0.06%; 95% confidence interval [CI], 0.06%–0.07%) had an ED presentation or hospitalisation with a code for elder abuse (Table 1). At the time of abuse, 137 people were home care package recipients, 112 were living in residential aged care and 333 were not receiving a home care package or living in residential aged care (of these 333,147 had previously accessed aged care, while 186 had no prior service use). Although rates of elder abuse captured during hospitalisations and ED presentations increased slightly over time, the annual incidence remained < 1% (Figures S1 and S2). State differences in elder abuse reporting are described in Tables S6 and S7.

**TABLE 1:** Cumulative incidence of elder abuse identified during hospitalisations and emergency department presentations among adults aged 65–105 years with aged care assessments (2010–2019, *n* = 965,986), overall and by type of aged care service received at the time of abuse.

Abuse type	All	Home care package	Residential aged care^a^	No aged care^b^
Any type of elder abuse				
Number of individuals	580	137	112	333
Cumulative incidence (95% CI)	0.06% (0.06%–0.07%)			
Physical				
Number of individuals	245	39	73	133
Cumulative incidence (95% CI)	0.03% (0.02%–0.03%)			
Neglect or abandonment				
Number of individuals	190	45	12	134
Cumulative incidence (95% CI)	0.02% (0.02%–0.02%)			
Sexual				
Number of individuals	25	< 6	18	< 6
Cumulative incidence (95% CI)	< 0.01%			
Psychological or emotional				
Number of individuals	69	27	< 6	39
Cumulative incidence (95% CI)	0.01% (0.01%–0.01%)			
Abuse, neglect and maltreatment without further specification				
Number of individuals	77	28	6	44
Cumulative incidence (95% CI)	0.01% (0.01%–0.01%)			
Domestic violence				
Number of individuals	295	83	13	201
Cumulative incidence (95% CI)	0.03% (0.03%–0.03%)			
Elder abuse or domestic violence				
Number of individuals	778	191	122	530
Cumulative incidence (95% CI)	0.08% (0.07%–0.09%)			

*Note:* Cells with counts less than 6 have been suppressed and reported as “< 6” to protect confidentiality. This table reports the number of unique individuals who experienced at least one episode of elder abuse or domestic violence across both hospitalisation and emergency department data. Individuals may have had multiple hospitalisations and accessed more than one type of aged care service over time. As a result, some categories in the table are not mutually exclusive.

Abbreviations: CI, confidence interval; ED, emergency department.

^a^ Residential respite care or permanent residential aged care.

^b^ Were not receiving a home care package or living in residential aged care at the time the abuse was recorded (147 had previously accessed aged care services, while 186 had no prior service use).

Of the total cohort, 245 individuals had a hospitalisation or ED presentation for physical abuse (0.03%; 95% CI, 0.02%–0.03%), 190 for neglect or abandonment (0.02%, 95% CI, 0.02%–0.02%), 77 for abuse, neglect and maltreatment without further specification (0.01%, 95% CI, 0.01%–0.01%), 69 for psychological or emotional abuse (0.01%, 95% CI, 0.01%–0.01%) and 25 for sexual abuse (< 0.01%).

The cumulative incidence of elder abuse was slightly higher in people living in socio‐economically disadvantaged areas (Socio‐Economic Indexes for Areas Index of Relative Socioeconomic Advantage and Disadvantage [SEIFA IRSAD] quintile 1 (least advantaged): 0.07%, 95% CI, 0.06%–0.09% versus SEIFA IRSAD quintile 5 (most advantaged): 0.05%, 95% CI, 0.04%–0.06%), for people living with dementia (0.08%, 95% CI, 0.07%–0.09% vs. no dementia: 0.05%, 95% CI, 0.05%–0.06%) and for people with a preferred language other than English (0.09%, 95% CI, 0.07%–0.11% vs. English: 0.06%, 95% CI, 0.05%–0.06%) (Table S8).

Overall, when also including codes for domestic violence, the incidence of elder abuse or domestic violence identified was 0.08% (778 people). There were 308 hospitalisations in 295 people (0.03%, 95% CI, 0.03%–0.03%) for domestic violence, of which, 24.7% (76) also had a code for elder abuse on the same day.

## 4 Discussion

The incidence of elder abuse identified in this study was 0.06% (580 people). This is consistent with findings from the United States, where estimates for elder abuse have been 0.05% based on hospitalisation data and 0.01% for ED visits [7, 8]. The estimate from our study is lower than elder abuse observed in other Australian studies (summary of studies shown in Table S9) [3, 9, 10]. The 2021 Australian National Elder Abuse Prevalence Study estimated that 14.8% of older Australians experienced elder abuse based on self‐reported data from a community survey [9]. By contrast, the 0.06% captured in this study reflects only cases recognised and coded during ED presentations or hospitalisations, capturing a subset of severe cases detected within acute care settings.

There are likely substantial under‐reporting and under‐recognition of elder abuse in hospital administrative data due to stigma, fear and lack of awareness, resulting in low sensitivity. Specificity may also vary by abuse subtype, with only the most severe cases captured, leading to underestimation of the true burden [11]. The World Health Organization (WHO) highlights the following as major barriers to the recognition and prioritisation of elder abuse globally: shame, stigmatisation, lack of awareness and complexity of each case, including the different types of abuse and variation by culture [11]. One of the five WHO priorities for tackling abuse of older people is improving data on prevalence and risk factors [11].

In recent years, growing awareness of family violence in Australia has led to improved legal, health and policy responses [4]. The aged care sector can build on these lessons to ensure elder abuse receives the same level of attention and action. Strengthening the identification and coding of elder abuse in Australian hospitals and EDs, and integration with other data sources, may improve detection and surveillance, contribute to global estimates and support population‐level strategies to reduce risk of harms. This could be achieved through clearer clinical guidelines and coding standards, routine screening and staff training in acute care settings, improved pathways for referral and reporting, and systematic linkage with aged care and healthcare datasets.

## Author Contributions

**Stephanie L. Harrison:** conceptualisation, methodology, project administration, supervision, interpretation of data, writing – original draft, and writing – review and edit. **Sahar Barmomanesh:** data analysis, interpretation of data, writing – original draft, and writing – review and edit. **Bryan Morden:** conceptualisation, methodology, interpretation of data, and writing – review and edit. **Maria C. Inacio:** conceptualisation, methodology, project administration, supervision, interpretation of data, and writing – review and edit. **Gillian E. Caughey:** conceptualisation, methodology, interpretation of data, and writing – review and edit.

## Funding

The Registry of Senior Australians (ROSA) Research Centre is supported through the Australian Government Medical Research Future Fund (PHRDI000009) and its partners (South Australian Health and Medical Research Institute, Caring Futures Institute, College of Nursing and Health Science at Flinders University, ECH Inc., Silverchain, Bolton Clarke). Prof Maria Inacio is supported by a National Health and Medical Research Council (NHMRC) Investigator Grant (GNT119378). Prof Gillian Caughey is supported by an NHMRC Investigator Grant (GNT2026400).

## Disclosure

Not commissioned; externally peer reviewed.

## Conflicts of Interest

The authors declare no conflicts of interest.

## Supporting information


**Data S1:** mja270172‐sup‐0001‐Supinfo.pdf.

## Data Availability

Data may be obtained from a third party and are not publicly available. The data for this study were obtained from the Australian Institute of Health and Welfare, Australian Government Department of Health and South Australia, Victoria, Queensland and New South Wales state health authorities and integrated by the Australian Institute of Health and Welfare, the NSW Centre for Health Record Linkage, the Centre for Victorian Data Linkage, Queensland Health's Statistical Services Branch and SA NT DataLink. These data were made available to the researchers under ethical, governance and confidentiality agreements that do not allow public sharing.
