# Supplementary material for: Emergency Department Presentations and Hospitalisations for Elder Abuse in People Accessing Aged Care Services in Australia: A Retrospective Cross‐Sectional Study
Source: Med J Aust. 2026 Mar 24;224(3):e70172. doi: 10.5694/mja2.70172 (PMC13010234; doi:10.5694/mja2.70172)
Supplement: Supplementary file 1 — Data S1: mja270172‐sup‐0001‐Supinfo.pdf. [file MJA2-224-0-s001.pdf]

Supporting Information

Supplementary material

This appendix was part of the submitted manuscript and has been peer reviewed.
It is posted as supplied by the authors.

Appendix to: Harrison SL, Barmomanesh S, Morden B, et al. Emergency department presentations and hospitalisations for elder abuse in people accessing aged care services in Australia: a retrospective cross-sectional study. *Med J Aust* 2026; doi: 10.5694/mja2.70172.

Supporting Information: Methods

Definition of hospitalisations with injuries related to domestic violence

Hospitalisations where the principal diagnosis was an injury (International Statistical Classification of Diseases and Related Health Problems, 10th revision, Australian Modification, [1] ICD-10-AM code range S00-T75, T79, Supporting Information, Table S2) and a first recorded external cause of morbidity was assault (ICD-10-AM code range X85-Y09, Supporting Information, Table S3), and a perpetrator coded as spouse or domestic partner, parent or other family member (5th character codes of 0,1,2 respectively).

This definition is based on the criteria used by the Australian Institute of Health and Welfare (AIHW) for identifying family and domestic violence-related hospitalisations [2].

Ascertainment of Indigenous status

Registry of Senior Australians (ROSA) data comprises multiple datasets from the National Aged Care Data Clearinghouse (NACDC) that identify Indigenous individuals. The datasets are weighted in order of priority (aged care service use, aged care assessment, commonwealth home support service use) and the highest priority non-missing value is taken. The completeness of this conciliated variable is at 76.7%. Individuals with a missing value for Indigenous status are not removed from the study.

Ascertainment of dementia status

Dementia status was ascertained using multiple administrative and clinical data sources. Evidence of dementia was identified from:
(i) health condition information recorded in aged care eligibility assessments;
(ii) the Rx-Risk-V comorbidity index derived from Pharmaceutical Benefits Scheme (PBS) dispensing records in the six months prior to assessment; and
(iii) hospital data using ICD-10-AM diagnostic codes recorded in admissions prior to assessment.
Dementia was defined by the presence of any of the following ICD-10-AM codes: F00*, F01*, F02*, F03*, F051*, G30*, G31.3, and U79.1 where the asterisk (*) denotes all subcategories.

Supporting Information Table S1. International Statistical Classification of Diseases and Related Health Problems, 10th revision, Australian Modification (ICD-10-AM) codes for elder abuse [1, 3]

Type of abuse	ICD-10 code	ICD Description
**Physical abuse**	T74.1	physical abuse, confirmed
	T76.1	physical abuse, suspected
	T74.11	adult physical abuse, confirmed
	T76.11	adult physical abuse, suspected
	Z04.7	encounter for examination and observation following alleged physical abuse
	Z04.71	encounter for examination and observation following alleged adult physical abuse
**Neglect or abandonment**	T74.0	neglect or abandonment, confirmed
	T76.0	neglect or abandonment, suspected
	T74.01	adult neglect or abandonment, confirmed
	T76.01	adult neglect or abandonment, suspected
**Sexual abuse**	T74.2	sexual abuse, confirmed
	T76.2	sexual abuse, suspected
	T74.21	adult sexual abuse, confirmed
	T76.21	adult sexual abuse, suspected
	T74.5	forced sexual exploitation, confirmed
	T76.5	forced sexual exploitation, suspected
	T74.51	adult forced sexual exploitation, confirmed
	T76.51	adult forced sexual exploitation, suspected
**Psychological or emotional abuse**	T74.3	psychological abuse, confirmed
	T76.3	psychological abuse, suspected
	T74.31	adult psychological abuse, confirmed
	T76.31	adult psychological abuse, suspected
**Abuse, neglect, and maltreatment without further specification**	Z69.11	counselling for victims of spousal and partner abuse
	T74.9	unspecified maltreatment, confirmed
	T76.9	unspecified maltreatment, suspected
	T74.91	unspecified adult maltreatment, confirmed
	T76.91	unspecified adult maltreatment, suspected
	T74	adult and child abuse, neglect and other maltreatment, confirmed
	T76	adult and child abuse, neglect and other maltreatment, suspected

Supporting Information Table S2. International Statistical Classification of Diseases and Related Health Problems, 10th revision, Australian Modification (ICD-10-AM) [1, 2] codes for injury-related principal diagnoses

ICD Description	ICD-10 range/code
Injuries to the head and face	S00*-S09*
Injuries to the neck	S10*-S19*
Injuries to the thorax	S20*-S29*
Injuries to the abdomen, lower back, pelvis, and lumbar spine	S30*-S39*
Injuries to the shoulder and upper arm	S40*-S49*
Injuries to the elbow and forearm	S50*-S59*
Injuries to the wrist, hand and fingers	S60*-S69*
Injuries to the femur, hip, and thigh	S70*-S79*
Injuries to the knee and lower leg	S80*-S89*
Injuries to the ankle and foot	S90*-S99*
Injuries involving multiple body regions	T00*-T06*
Unspecified multiple injuries	T07
Injuries to the spine, trunk, and limbs with unspecified level	T08*-T13*
Injuries to unspecified body regions	T14*
Injuries from foreign bodies entering through natural orifices	T15*-T19*
Burns	T20*-T31*
Frostbite	T33*-T34*
Frostbite involving multiple body regions and unspecified frostbite	T35*
Poisoning by drugs or biological substances	T36*-T50*
Toxic effects of alcohol and nonmedicinal substances	T51*-T65*
Other and unspecified effects due to radiation, heat, hypothermia, and cold	T66*-T69*
Effects of air pressure and water pressure	T70*
Asphyxiation	T71
Effects of deprivation	T73*
Maltreatment syndromes	T74*
Effects of other external causes	T75*
Early complications of trauma, not elsewhere classified	T79*

Supporting Information Table S3. International Statistical Classification of Diseases and Related Health Problems, 10th Revision, Australian Modification (ICD-10-AM) [1, 2] codes for external cause of morbidity related to family violence-related assaults

ICD Description	ICD-10 code*
Assault by drugs, medicaments and biological substances	X85
Assault by corrosive substance	X86
Assault by pesticides	X87
Assault by gases and vapours	X88
Assault by other specified chemicals and noxious substances	X89
Assault by unspecified chemical or noxious substance	X90
Assault by hanging strangulation and suffocation	X91
Assault by drowning and submersion	X92
Assault by handgun discharge	X93
Assault by rifle, shotgun and larger firearm discharge	X94
Assault by other and unspecified firearm discharge	X95
Assault by explosive material	X96
Assault by smoke, fire and flames	X97
Assault by steam, hot vapours and hot objects	X98
Assault by sharp object	X99
Assault by blunt object	Y00
Assault by pushing from a high place	Y01
Assault by crashing of motor vehicle	Y02
Assault by other means of pushing from high place	Y03
Assault by bodily force	Y04
Sexual assault by bodily force	Y05
Neglect and abandonment	Y06
Other maltreatment syndromes	Y07
Assault by other specified means	Y08
Assault by unspecified means	Y09

* Only codes with extensions .00, .01, and .02 following each main code were included. These represent assaults perpetrated by a spouse or domestic partner (.00), a parent (.01), or another family member (.02), respectively.

Supporting Information Table S4. Strengthening the Reporting of Observational Studies in Epidemiology (STROBE) statement checklist for cross-sectional studies

	Item No	Recommendation	Page No
**Title and abstract**	1	(*a*) Indicate the study's design with a commonly used term in the title or the abstract	1
		(*b*) Provide in the abstract an informative and balanced summary of what was done and what was found	1
**Introduction**			
Background/rationale	2	Explain the scientific background and rationale for the investigation being reported	1
Objectives	3	State specific objectives, including any prespecified hypotheses	1
**Methods**			
Study design	4	Present key elements of study design early in the paper	1
Setting	5	Describe the setting, locations, and relevant dates, including periods of recruitment, exposure, follow-up, and data collection	1
Participants	6	(*a*) Give the eligibility criteria, and the sources and methods of selection of participants	1
Variables	7	Clearly define all outcomes, exposures, predictors, potential confounders, and effect modifiers. Give diagnostic criteria, if applicable	1, Table S4
Data sources/ measurement	8*	For each variable of interest, give sources of data and details of methods of assessment (measurement). Describe comparability of assessment methods if there is more than one group	1, Supplementary Methods, Table S1, Table S2, Table S3
Bias	9	Describe any efforts to address potential sources of bias	N/A
Study size	10	Explain how the study size was arrived at	N/A
Quantitative variables	11	Explain how quantitative variables were handled in the analyses. If applicable, describe which groupings were chosen and why	1, Supplementary Methods, Table S1, Table S2, Table S3
Statistical methods	12	(*a*) Describe all statistical methods, including those used to control for confounding	1
		(*b*) Describe any methods used to examine subgroups and interactions	N/A
		(*c*) Explain how missing data were addressed	N/A
		(*d*) If applicable, describe analytical methods taking account of sampling strategy	N/A
		(*e*) Describe any sensitivity analyses	N/A
**Results**			
Participants	13*	(a) Report numbers of individuals at each stage of study—eg numbers potentially eligible, examined for eligibility, confirmed eligible, included in the study, completing follow-up, and analysed	1, 2
		(b) Give reasons for non-participation at each stage	N/A
		(c) Consider use of a flow diagram	N/A
Descriptive data	14*	(a) Give characteristics of study participants (eg demographic, clinical, social) and information on exposures and potential confounders	2, Table S4
		(b) Indicate number of participants with missing data for each variable of interest	N/A
Outcome data	15*	Report numbers of outcome events or summary measures	Table 1, Table S5 Table S6, Table S7
Main results	16	(*a*) Give unadjusted estimates and, if applicable, confounder-adjusted estimates and their precision (eg, 95% confidence interval). Make clear which confounders were adjusted for and why they were included	Table 1, Table S5 Table S6, Table S7, Figure S1, Figure S2
		(*b*) Report category boundaries when continuous variables were categorized	N/A
		(*c*) If relevant, consider translating estimates of relative risk into absolute risk for a meaningful time period	N/A
Other analyses	17	Report other analyses done—eg analyses of subgroups and interactions, and sensitivity analyses	N/A

**Discussion**			
Key results	18	Summarise key results with reference to study objectives	2, 3
Limitations	19	Discuss limitations of the study, taking into account sources of potential bias or imprecision. Discuss both direction and magnitude of any potential bias	3
Interpretation	20	Give a cautious overall interpretation of results considering objectives, limitations, multiplicity of analyses, results from similar studies, and other relevant evidence	2, 3
Generalisability	21	Discuss the generalisability (external validity) of the study results	2, 3
**Other information**			
Funding	22	Give the source of funding and the role of the funders for the present study and, if applicable, for the original study on which the present article is based	3

*Give information separately for exposed and unexposed groups.

Note: An Explanation and Elaboration article discusses each checklist item and gives methodological background and published examples of transparent reporting. The STROBE checklist is best used in conjunction with this article (freely available on the Web sites of PLoS Medicine at http://www.plosmedicine.org/, Annals of Internal Medicine at http://www.annals.org/, and Epidemiology at http://www.epidem.com/). Information on the STROBE Initiative is available at www.strobe-statement.org.

Supporting Information Table S5. Characteristics of the study cohort at the time of aged care eligibility assessment (2010–2019; n=965,986), overall and by type of aged care service received in the study period (home care package, residential aged care, or no home or residential care)

Characteristic	Study cohort	Home care package recipients	Residential aged care recipients	No home care or residential aged care
**Total**, N	**965,986**	**263,603**	**575,926**	**274,543**
**Age (years)**, median (interquartile range)	83 (77-88)	82 (76-87)	84 (79-88)	81 (75-86)
**Age categories (years)**, % (n)				
65-74	17.2 (166,558)	19.2 (50,636)	13.3 (76,822)	22.2 (60,897)
75-84	41.6 (401,445)	44.9 (118,342)	39.7 (228,791)	43.5 (119,367)
≥85	41.2 (397,983)	35.9 (94,625)	46.9 (270,313)	34.3 (94,279)
**Female***, % (n)	59.1 (571,139)	63.3 (166,946)	60.0 (345,679)	55.9 (153,418)
**Preferred language other than English**, % (n)	10.7 (103,356)	12.5 (32,981)	9.4 (54,261)	11.2 (30,707)
Missing	2.1 (19,889)	0.6 (1,558)	0.4 (2,431)	5.9 (16,207)
**Country of birth not Australia**, % (n)	32.3 (311,875)	34.8 (91,712)	30.4 (175,179)	33.7 (92,580)
Missing	0.5 (4,874)	0.1 (278)	0.2 (1,394)	1.2 (3,267)
**Remoteness**, % (n)				
Major cities	67.3 (650,511)	66.5 (175,412)	68.3 (393,425)	65.7 (180,255)
Inner Regional	23.5 (227,368)	24.6 (64,833)	23.3 (133,912)	23.8 (65,206)
Outer Regional	8.3 (80,189)	8.1 (21,356)	7.8 (45,091)	9.4 (25,788)
Remote	0.5 (5,057)	0.5 (1,206)	0.4 (2,109)	0.8 (2,279)
Very Remote	0.2 (1,608)	0.2 (480)	0.1 (596)	0.3 (694)
Missing	0.1 (1,253)	0.1 (316)	0.1 (793)	0.1 (321)
**State**, % (n)				
New South Wales	39.0 (377,101)	40.7 (107,195)	39.7 (228,863)	37.1 (101,953)
Victoria	30.5 (295,026)	26.8 (70,650)	29.8 (171,896)	33.1 (90,739)
Queensland	20.5 (197,785)	23.0 (60,623)	19.8 (113,843)	20.6 (56,490)
South Australia	9.9 (96,074)	9.5 (25,135)	10.6 (61,324)	9.2 (25,361)
**SEIFA IRSAD quintile**, % (n)				
1 (least advantaged)	19.4 (187,262)	19.5 (51,336)	19.0 (109,500)	19.8 (54,269)
2	18.9 (182,091)	19.2 (50,627)	18.5 (106,602)	19.2 (52,832)
3	18.9 (182,336)	19.6 (51,748)	18.5 (106,743)	19.2 (52,671)
4	18.5 (178,903)	18.4 (48,508)	18.7 (107,840)	18.2 (49,923)
5 (most advantaged)	24.2 (234,179)	23.2 (61,099)	25.1 (144,484)	23.5 (64,519)
Missing	0.1 (1,215)	0.1 (285)	0.1 (757)	0.1 (329)
**Living arrangements**, % (n)				
Lives alone	43.4 (419,201)	44.3 (116,814)	46.5 (267,546)	38.0 (104,409)
Lives with family/partner	53.0 (511,618)	53.5 (141,080)	49.0 (282,072)	59.6 (163,589)
Lives with others	2.0 (18,947)	1.7 (4,465)	2.1 (12,114)	1.8 (4,879)
Missing/not applicable/not specified	1.7 (16,220)	0.5 (1,244)	2.5 (14,194)	0.6 (1,666)
**Informal carer support**, % (n)	79.4 (767,206)	79.5 (209,586)	82.1 (472,575)	75.8 (208,042)
Missing/not applicable/not specified	2.5 (24,068)	1.3 (3,316)	2.5 (14,262)	2.8 (7,801)
**Dementia diagnosis**, % (n)	21.8 (210,713)	20.0 (52,606)	28.2 (162,604)	12.6 (34,718)

Abbreviations: SEIFA IRSAD = Socioeconomic Indexed for Areas Index of Relative Socioeconomic Advantage and Disadvantage.
Categories are not mutually exclusive i.e., a participant may have accessed home care and residential aged care in the study period.
*Sex was recorded as female or male and reflects sex assigned at birth; information on gender identity was not available.

Supporting Information Table S6. Frequency of elder abuse types coded during inpatient hospitalisations, by state, 2010–2019 (overall; not disaggregated by type of aged care service received)

Abuse type	All	NSW	VIC	QLD	SA
**Total inpatient hospitalisations,** N	**5,033,129**	**1,937,529**	**1,570,391**	**1,211,675**	**313,534**
**Physical**					
Number of hospitalisations	123	52	25	33	13
Rate per 1000 hospitalisations (95% CI)	0.02 (0.02-0.03)	0.03 (0.02-0.04)	0.02 (0.01-0.02)	0.03 (0.02-0.04)	0.04 (0.02-0.07)
**Neglect or abandonment**					
Number of hospitalisations	125	47	23	45	10
Rate per 1000 hospitalisations (95% CI)	0.02 (0.02-0.03)	0.02 (0.02-0.03)	0.01 (0.01-0.02)	0.04 (0.03-0.05)	0.03 (0.02-0.06)
**Sexual**					
Number of hospitalisations	11	<6	<6	<6	<6
Rate per 1000 hospitalisations (95% CI)	<0.01				
**Psychological or emotional**					
Number of hospitalisations	67	34	12	14	7
Rate per 1000 hospitalisations (95% CI)	0.01 (0.01-0.02)	0.02 (0.01-0.02)	0.01 (<0.01-0.01)	0.01 (0.01-0.02)	0.02 (0.01-0.05)
**Abuse, neglect and maltreatment without further specification**					
Number of hospitalisations	26	7	11	8	0
Rate per 1000 hospitalisations (95% CI)	0.01 (<0.01-0.01)	<0.01 (<0.01-0.01)	0.01 (<0.01-0.01)	0.01 (<0.01-0.01)	0
**Multiple type of abuse**					
Number of hospitalisations	17	10	<6	<6	<6
Rate per 1000 hospitalisations (95% CI)	<0.01	0.01 (<0.01-0.01)			
**Any elder abuse**					
Number of hospitalisations	333	130	74	101	28
Rate per 1000 hospitalisations (95% CI)	0.07 (0.06-0.07)	0.07 (0.06-0.08)	0.05 (0.04-0.06)	0.08 (0.07-0.10)	0.09 (0.06-0.13)

Abbreviations: CI = confidence interval; NSW = New South Wales; QLD = Queensland; SA = South Australia; VIC = Victoria.

Cells with counts less than 6 have been suppressed and reported as "<6" to protect confidentiality.

Counts represent episodes of care rather than unique individuals. Emergency department presentations and inpatient hospitalisations were analysed separately, and individuals may contribute multiple episodes across the study period.

Supporting Information Table S7. Frequency of elder abuse types coded during emergency department presentations, by state, 2010–2019 (overall; not disaggregated by type of aged care service received)

Abuse type	All	NSW	VIC	QLD	SA
**Total emergency department presentations**, N	**2,877,536**	**1,274,887**	**764,238**	**614,357**	**224,054**
**Physical**					
Number of ED presentations	128	117	0	10	<6
Rate per 1000 ED presentations (95% CI)	0.04 (0.04-0.05)	0.09 (0.08-0.11)		0.02 (0.01-0.03)	
**Neglect or abandonment**					
Number of ED presentations	74	54	0	<6	18
Rate per 1000 ED presentations (95% CI)	0.03 (0.02-0.03)	0.04 (0.03-0.06)			0.08 (0.05-0.13)
**Sexual**					
Number of ED presentations	*	*	0	8	0
Rate per 1000 ED presentations (95% CI)	*	*		0.01 (0.01-0.03)	
**Psychological or emotional**					
Number of ED presentations	<6	<6	0	<6	0
Rate per 1000 ED presentations (95% CI)					
**Abuse, neglect and maltreatment without further specification**					
Number of ED presentations	56	19	31	<6	<6
Rate per 1000 ED presentations (95% CI)	0.02 (0.01-0.03)	0.01 (0.01-0.02)	0.04 (0.03-0.06)		
**Any elder abuse**					
Number of ED presentations	277	200	31	24	22
Rate per 1000 ED presentations (95% CI)	0.10 (0.09-0.11)	0.16 (0.14-0.18)	0.04 (0.03-0.06)	0.04 (0.03-0.06)	0.10 (0.06-0.15)

Abbreviations: ED = Emergency department; CI = confidence interval; NSW = New South Wales; QLD = Queensland; SA = South Australia; VIC = Victoria.
Cells with counts less than 6 have been suppressed and reported as "<6" to protect confidentiality.
Counts represent episodes of care rather than unique individuals. Emergency department presentations and inpatient hospitalisations were analysed separately, and individuals may contribute multiple episodes across the study period.
* The second smallest value in each column was masked to prevent disclosure of a cell with a count less than 6.

Supporting Information Table S8. Cumulative incidence of elder abuse identified during hospitalisations and emergency department presentations among adults aged 65–105 years with aged care eligibility assessments (2010–2019, n=965,986), by participant characteristics

	Number of individuals	Number of individuals with elder abuse*	Cumulative incidence (95% CI)
**Informal carer support**			
Yes	767,206	461	0.06 (0.05-0.07)
No	174,712	110	0.06 (0.05-0.08)
**SEIFA IRSAD quintile**			
1 (least advantaged)	187,262	140	0.07 (0.06-0.09)
2	182,091	108	0.06 (0.05-0.07)
3	182,336	109	0.06 (0.05-0.07)
4	178,903	104	0.06 (0.05-0.07)
5 (most advantaged)	234,179	115	0.05 (0.04-0.06)
**Remoteness**			
Major cities	650,511	401	0.06 (0.06-0.07)
Regional	307,557	169	0.05 (0.05-0.06)
Remote/very remote	6,665	6	0.09 (0.03-0.20)
**Preferred language**			
English	842,741	486	0.06 (0.05-0.06)
Other than English	103,356	91	0.09 (0.07-0.11)
**Dementia diagnosis**			
Yes	210,713	166	0.08 (0.07-0.09)
No	755,273	414	0.05 (0.05-0.06)

Abbreviations: CI = confidence interval; SEIFA IRSAD = Socio-Economic Indexes for Areas Index of Relative Socioeconomic Advantage and Disadvantage.
*With any code for elder abuse during a hospitalisation or emergency department presentation after aged care assessment.

Supporting Information Table S9. Findings from this study and previous national studies

Measure	ROSA	NEAPS	SIRS*	OPAN ±
**Reporting source**	ROSA National Historical Cohort, N=965,986 non-Indigenous individuals aged 65+ years assessed for aged care eligibility in SA, NSW, QLD, and VIC in 2010–2019.	National survey, N=7,000 community-dwelling people aged 65+ years (2020), excluding residential aged care residents in 2020.	Government-mandated surveillance under the Serious Incident Response Scheme (SIRS) for the period 2023–2024, based on incident reports submitted by aged care providers.	Passive surveillance by OPAN network members, based on N=44,000 advocacy and information requests from older people, families, and representatives in 2023–2024.
**Setting**	Residential aged care (respite or permanent) and at home (with or without receiving home care services).	At home (with or without receiving home care services).	Residential aged care and at home receiving home care services.	Residential aged care and at home (with or without receiving home care services).
**Elder abuse incidents by setting (n)**				
Any	580 (778 including domestic violence)	1,050	61,960	3,125
At home (with or without receiving home care services)	-	1,050	-	-
At home receiving home care services	136	-	5,005	-
At home without receiving home care services	330	-	-	
Residential aged care	116	-	56,955	-
**Elder abuse incidence by type (% or rate)**				
Any type of abuse	0.06% (0.08% including domestic violence)	15%	7.9	7%
Physical	0.03%	2%	4.4	-
Psychological or emotional	0.01%	12%	0.72	0.45%
Neglect or abandonment	0.02%	3%	1.9	-
Sexual	<0.01%	1%	0.3	-
Other types of elder abuse	Abuse, neglect and maltreatment without further specification: 0.01%		Inappropriate use of restrictive practices: 0.1<br>Stealing or financial coercion: 0.1	Financial abuse: 0.48%<br>Substitute decision-maker issues: 0.48%<br>Family conflict: 0.40%<br>Coercive control: 0.33%

Abbreviations: ROSA = Registry of Senior Australians; NEAPS = National Elder Abuse Prevalence study; SIRS = Serious Incident Response Scheme; OPAN = Older Persons Advocacy Network.
± Network members provided 3,125 services, 2,502 information and 623 advocacies, related to abuse of older people by individuals other than aged care workers. Percentages for types of elder abuse were calculated by dividing the number of reported issues by the total number of requests received (n=44,000).
* Rates reported for SIRS are for residential care only. In the SIRS report, abuse rates are calculated based on the number of incidents reported each quarter, standardised per 10,000 occupied bed days (OBDs) in residential care. Although incident counts for home services are provided in the Sector Performance Report (Q1 2024–25), no standardised rate (e.g., per 10,000 OBDs) is reported, and therefore these data are not included.
These studies are provided for contextual purposes only.

Supporting Information Figure S1. Annual rates of emergency department presentations and hospitalisations with coded elder abuse per 1000 emergency department presentations or hospitalisations, 2011–2019

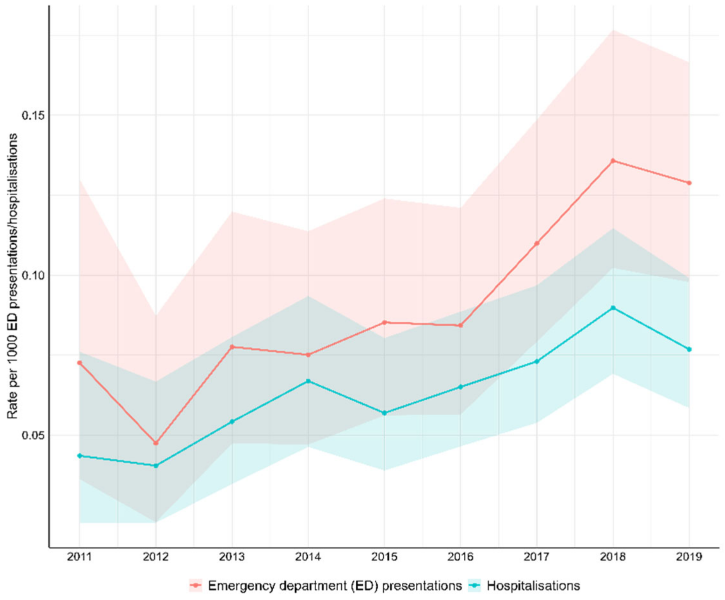

The year 2010 was excluded due to small counts (<6) to protect confidentiality.
Domestic violence-related codes were not included.
Shaded areas represent 95% confidence intervals.

Supporting Information Figure S2. Annual incidence of coded elder abuse among individuals presented to emergency departments or hospitalised, 2011–2019.

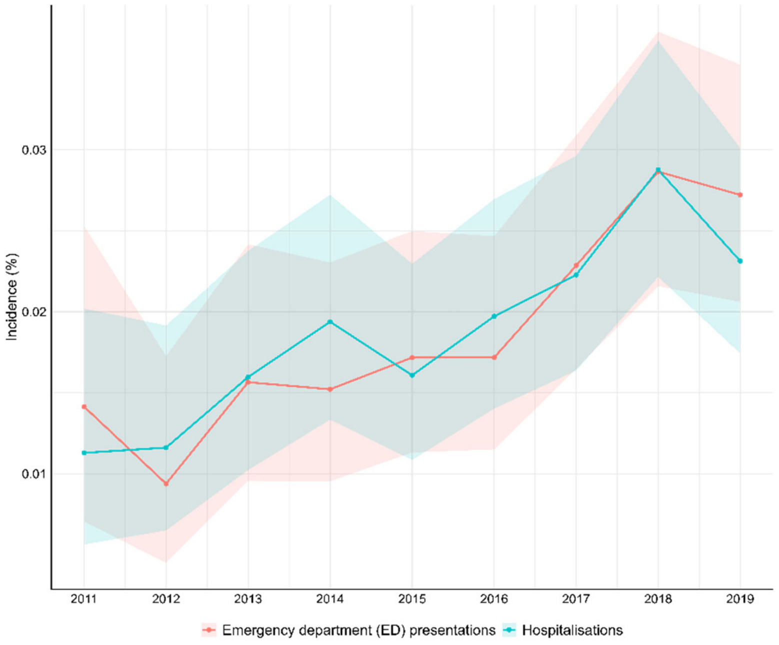

The year 2010 was excluded due to small counts (<6) to protect confidentiality.
Domestic violence-related codes were not included.
Shaded areas represent 95% confidence intervals.

References

1. Independent Health and Aged Care Pricing Authority, The International Statistical Classification of Diseases and Related Health Problems, Tenth Revision, Australian Modification (ICD-10-AM), Australian Classification of Health Interventions (ACHI) and Australian Coding Standards (ACS) (ICD-10-AM/ACHI/ACS), 11th ed. (Darlinghurst, NSW: Independent Health and Aged Care Pricing Authority, 2022).

2. Australian Institute of Health and Welfare, Family, Domestic and Sexual Violence (AIHW, 2024), https://www.aihw.gov.au/family-domestic-and-sexual-violence/responses-and-outcomes/health-services (viewed December 2024).

3. T. Rosen, K. Wen, LK Makaroun, et al., "Diagnostic Coding of Elder Mistreatment: Results from a National Database of Medicare Advantage and Private Insurance Patients, 2011–2017," Journal of Applied Gerontology 41, no. 4 (2022): 918–927.
